# The effect of social group size on feather corticosterone in the co-operatively breeding Smooth-billed Ani (*Crotophaga ani*): An assay validation and analysis of extreme social living

**DOI:** 10.1371/journal.pone.0174650

**Published:** 2017-03-29

**Authors:** Joshua K. Robertson, Cameron Muir, Conner S. Hurd, Jing S. Hing, James S. Quinn

**Affiliations:** 1 Department of Biology, McMaster University, Hamilton, Ontario, Canada; 2 Department of Psychology, Centre for Neuroscience, Brock University, Saint Catherines, Ontario, Canada; Columbia University, UNITED STATES

## Abstract

Living closely with others can provide a myriad of fitness benefits, from shared territory defense to co-operative resource acquisition. Costs of social aggregation are not absent, however, and likely influence optimal and observed groups’ sizes in a social species. Here, we explored optimal group size in a joint-nesting cuckoo species (the Smooth-billed Ani, *Crotophaga ani*) using endocrine markers of stress physiology (corticosterone, or CORT). Smooth-billed Anis exhibit intense reproductive competition that is exacerbated in atypically large groups. We therefore hypothesized that intra-group competition (measured by social group size) mediates the desirability and physiological cost of social group membership in this species. To test this hypothesis, we captured 47 adult Smooth-billed Anis (31 males, 16 females) during the breeding seasons of 2012-2014 in south-western Puerto Rico, and documented social group sizes. Tail feathers were sampled and used to quantify CORT (pg/mg) in enzyme-linked immunosorbent assays (ELISAs) (n = 50). Our analyses show significant differences in feather-CORT of adults between categorical group sizes, with individuals from atypically large social groups (≥ *x* + 1SD) having highest mean concentrations (33.319 pg/mg), and individuals from atypically small social groups (≤ *x* − 1SD) having lowest mean concentrations (8.969 pg/mg). Whether reproductive competition or effort is responsible for elevated CORT in atypically large social groups, however, remains unclear. Our results suggest that living in atypically large groups is physiologically expensive and may represent an evolutionarily unstable strategy. To our knowledge, this is the first study to explore a correlation between stress physiology and group size in a joint-nesting species.

## Introduction

Living in groups can provide numerous fitness benefits, either directly (through shared territory defense, predator defense, resource acquisition, and parental effort) (for example, [1–8]) or indirectly (through dilution of predation risk and inclusive fitness benefits) [9, 10]. Costs of social aggregation, however, are not absent and are thought to influence optimal and realized (or observable) group sizes in a gregarious species [11–14]. Indeed, theoretical research suggests that realized group sizes can be explained by net fitness benefits that are outweighed by costs when group membership is atypically high, or lost when membership is atypically low [11–13]. Where social groups are atypically large, competition for access to resources and mating opportunities, frequent agonistic interactions and heightened spread of disease may limit incentives to join, or remain in social groups [14–17]. In atypically small groups, advantages of group living such as shared territory and predator defense are diminished and provide incentives to join larger social clusters [11]. Among co-operative breeders (particularly those with multiple breeding males and females), competition for reproductive opportunities and benefits of shared parental effort are strong antagonistic forces that likely influence optimal group size. Facultative co-operative breeders, therefore, provide an ideal framework to investigate the costs and benefits of group formation and optimal group size theory.

Across vertebrates, activation of the hypothalamic-pituitary-adrenal (HPA) axis is widely used as a indicator of stress [18]. HPA activation in response to perceived stressors results in the secretion of glucocorticoids from the adrenal cortex, which mobilizes energy stores through lipolysis, gluconeogenesis and proteolysis [19, 20]. Temporary elevation of serum glucocorticoids is therefore thought to act adaptively by routing energy stores to solve immediate problems. Supporting this theory, temporary elevation of serum corticosterone (or ‘CORT’, the primary glucocorticoid in birds) has exhibited positive correlations with cognition [21–23], caching behaviour [23–25] and survivorship [26, 27]. Chronic HPA activation, however, presents considerable metabolic demands and can yield detrimental effects on reproduction and survival [19, 28–31]. While numerous studies have examined the effects of social hierarchy on circulating CORT titers in birds [25, 32, 33], few have investigated the influence of social group size on stress physiology [17, 34] and fewer still in joint-nesting species [34].

Smooth-billed Anis (*Crotophaga ani*) are neotropical cuckoos that form socially breeding groups of monogomous pairs during the breeding season. All social group members share in nest building and care of young. Competition for maternal representation in communal nests is intense and costs associated with compensation for egg losses by tossing and burial are thought to be high [35]. Schmaltz et al [35], for example, reported a negative correlation between per capita reproductive success and group size, with egg loss per capita also increasing with maternal group membership. Despite these reproductive losses, direct benefits of group living, such as alarm calling [36], shared provisioning, and shared territory defense [37] have been reported in this species and may provide incentives for joining or establishing socially breeding groups. Alternatively, solitary nesting is not uncommon and presents an opportunity to capitalize on genetic contribution to a brood, though direct benefits of group living must be forgone. Trends of social group size and CORT deposited in yolk have been explored in Smooth-billed Ani eggs [34] and have elucidated a positive correlation between maternal group size and CORT deposition. Similar analyses, however, are lacking for breeding adults and may shed light on the desirability of grouping behaviour and social group maintenance.

Here, we tested the relationship between social group size and CORT deposited in feathers of joint-nesting Smooth-billed Anis, grown in the breeding season. Feather-CORT is thought to reflect plasma titers at the time of feather growth and therefore provide a stable, time-averaged archive of baseline and stress-induce CORT in birds [38, 39]. First, we validated an enzyme-linked immunosorbent assay (ELISA) to detect and quantify CORT deposited in rectrices. We then leveraged this ELISA to analyze trends of feather-CORT and social group size. We hypothesized that intra-group competition mediates stress physiology and the desirability of social group membership in adult Anis. We therefore predicted that; 1) feather-CORT is highest in large social groups where social competition is highest, and 2) feather-CORT differs between sexes in adults according to intensity of reproductive competition. Previous research posits that sexual selection may be acting on male bill size [40], implying intra-male competition for breeding opportunities. Intra-male competition is likely to be costly [41] and may promote heightened circulating and feather deposition of CORT in adult males. Alternatively, females may exhibit higher feather-CORT than males due to heightened costs of egg loss by ovicide and reproductive compensation. By analyzing trends of time-average HPA activation across adult Anis, we aim to illuminate the physiological consequences of social grouping in a joint-nesting, breeding framework which are yet to be clarified in literature.

## Materials and methods

### Field location

Wild Smooth-billed Anis were captured and observed at the Cabo Rojo National Wildlife Refuge (NWR) and immediately surrounding private land in south-western Puerto Rico, from 2012-2014. The habitat of the Cabo Rojo NWR is dry forest and pasture land with discrete, annual wet and dry seasons. Smooth-billed Anis inhabit the wildlife refuge year-round, though breeding is limited to the wet season when invertebrate prey is abundant (September to December). All capture and observations were limited to breeding seasons, as we were interested in effects of reproductive competition on stress in adults.

### Adult capture and sampling

All capture and sampling procedures (mist-netting, trapping, banding, blood sampling and rectrix sampling) were conducted under licensees from the United States Fish and Wildlife Service (USFWS) with ethical review and approval from the McMaster University Animal Care Committee (AUP 13-10-37). Land use permission for capture and observations were acquired from the UFSWS (Cabo Rojo NWR) and private land owners.

Adults were primarily captured using two horizontally stacked mist-nests (6.6 cm mesh, 18 m long, 7.2 m high) on telescopic poles [42, 43] as they left night roosts. We also captured adults using funnel traps, “baited” with a hand-raised, conspecific lure bird in a protective cage [44], and using hardware cloth, walk-in nest traps [45]. Once captured, we collected 50 μl to 200 μl of blood from each individual for molecular sexing. Blood was sampled by brachial or basilic venipuncture and capillary tube collection, stored in a small volume (approximately 1 mL) of Queen’s Lysis Buffer [46], and kept at 4°C until required for genomic DNA isolation.

Immediately following blood-sampling, we plucked the left, outermost rectrix (R4) of each individual, and stored them in sealed plastic bags until use in endocrine analyses. Though molt patterns are not definitive for Smooth-billed Anis, previous research [47] suggests an overlap between breeding season and molt; a pattern that is reportedly common among tropical birds [48]. Unpublished observations (Parks M, Robertson JK & Smith N) of molting, adult Smooth-billed Anis captured during the beginning of the breeding season (September of 2015) support observations of molting individuals in the breeding season by Snow and Snow [47]. Newly grown feathers are readily identifiable by an iridescence which fades to matte brown over time [49] (Quinn, JS, Parks M, Robertson JK & Smith N, personal observations) (S1 Fig). We therefore only included data from individuals with iridescent, new feathers. Data from feathers exhibiting slight fading (n = 7) were also included in statistical analyses to bolster sample size, but were removed in supplementary analyses to confirm reported trends (S1 Methods; S1 and S2 Tables).

All captured adults were assigned a unique combination of three Darvic coloured leg bands and one USGS numbered aluminum band for future identification and released to their respective home-territories.

For this study, a total of 47 adult Anis were captured, sampled and banded. Of the 47 adults, three were recaptured at subsequent trapping events (n = 2 recaptured during the 2014 breeding season, and n = 1 recaptured across two consecutive breeding seasons). In the event of recapture, the left, regrown R4 was plucked as described previously. Recapture samples were included in analyses to individual ID accounted for in statistical analyses.

### Social group size determination

Social group sizes were estimated by counting the number of individuals leaving from, and returning to communal roosts [43]. Juveniles are easily recognized and excluded from group size counts.

All 47 sampled adults were captured from 30 social groups ranging from 3 to 13 individuals, with a mean membership of 7.04 individuals (± SD = 3.36) across all years. For statistical analyses, group sizes were categorized according to their deviation from mean membership. Group sizes exceeding mean membership plus one standard deviation (≥ *x* + 1SD = 10) were considered atypically large, while those below one standard deviation from the mean were considered atypically small (≤ *x* − 1SD = 3). Of the 30 social groups observed, 4 were categorized as atypically small, 21 as intermediately sized and 5 as atypically large. Mean group sizes of atypically small, intermediate and atypically large social groups were 3.00 (± SD = 0 (n = 7), 5.64 (± SD = 1.47 (n = 27) and 11.53 (± SD = 1.30 (n = 13) respectively.

### Molecular sex determination

Genomic DNA was isolated from blood-lysis samples by phenol:chloroform extraction and precipitation with isopropyl alcohol, then stored in TE (Tris-EDTA, pH = 7.4) buffer at -20°C until further use. Smooth-billed Anis exhibit sexual dimorphism in bill and body size, however, overlap is considerable (Robertson, 2016) and limits accurate sex determination by morphology. We therefore determined the sex of all captured adults by PCR amplification of the Chromo-Helicase DNA binding (CHD) gene [50, 51] and amplicon size separation on 1-3% agarose gels. For this study, rectrices were collected from 16 females and 31 males. Group size categories (atypically small, intermediate and atypically large) contained n_Males_ = 5, n_Females_ = 2; n_Males_ = 20, n_Females_ = 7; and n_Males_ = 6, n_Females_ = 7 respectively.

### Steroid hormone extractions

Extraction of CORT from rectrices followed protocols by Bortolotti et al [38], with slight modifications. Briefly, rectrix samples were washed sequentially under pure water and 100% methanol to remove fecal contamination and to strip preen oils. The calamus was then removed to eliminate contamination by dried blood. Once dried, we sampled a 70-80 mm section of each rectrix, measured from the proximal end of the feather. Standardization of section length was used to account for observed biases in feather-CORT according to feather size [52]. Samples were then diced into 5 x 5 mm sections and weighed in 25 mL sterile scintillation vials to the nearest tenth of a milligram. To each sample, we added 1 mL of 100% methanol for every 5 mg of rectrix fragments prior to incubation at 50°C for 24 hours. Methanol extracts were then sterile filtered using 0.22 μm syringe filters into new scintillation vials. Feather fragments were vortexed in 1 mL of diethyl ether for 30 seconds, and ether extractions were sterile filtered and pooled with methanol extracts [31]. All samples were stored at -20°C until further use. Prior to analysis, we evaporated methanol-ether extracts under a fume hood at room temperature until dry and reconstituted steroid samples to the appropriate volume in phosphate assay buffer (pH 7.0) (approximately 20x concentration).

### Enzyme-linked immunosorbent assays

We quantified CORT in feather isolates using ELISAs. All assays were conducted using NUNC Maxisorp plates and a CORT antibody (Esoterix B3-163, Calabasas Hills, California, USA) shown to have low cortisol (0.4%) and cortisone (0.1%) cross-reactivity [53]. CORT standard was obtained from Steraloids Inc. (Newport, Rhode Island, USA) and a horse-radish peroxidase (HRP) conjugate was obtained from Creative Diagnostics Inc. (Shirley, New York, USA). NUNC plates were first coated with 50 μl of anti-CORT diluted to 1:10,000 in coating buffer (50 mM bicarbonate buffer, pH = 9.6) and incubated at 4°C for 12 hours. Wells were then washed five times with 100 μl of a Tween wash solution (0.15 M NaCl and 0.5 mL Tween 20/L) to remove excess, unbound antibody and incubated at room temperature for 2 hours with 50 μl of phosphate assay buffer. Following incubation, assay buffer was removed and CORT standard, samples and HRP conjugate were added to each well. Here, 50 μl of standard or sample were added to each well, followed by 50 μl of conjugate, diluted to 1:10,000 from a 1 mg/mL stock in assay buffer. On each plate, we used a twelve-point standard curve ranging from 5,000 pg/well to 2.44 pg/well of CORT standard. All samples and standards were run in duplicate. Following addition of samples, conjugate and CORT standard, plates were left to incubate for 1.5 hours, then washed again with Tween wash solution to remove unbound HRP-conjugate. Next, 100 μl of a substrate solution containing citrate buffer, H_2_O_2_ and 2,2’-azine-bis [3-ethylbenzthiazoline-6-sulfonic acid] were added to each well and plates were shaken until standard curves were maximized (difference between the minimum and maximum optical density (OD) in standard curve was greatest; between 1 and 2 hours). The OD at 405 nm of wells was then measured using a microplate reader and a log-log regression line was plotted to the standard curve (log OD vs. log CORT (pg/well)). Mass of CORT per well was quantified by interpolation from the standard curve. Only samples with a coefficient of variation (CV) less than 15% between duplicates were selected for use in statistical analyses.

Though Bortolotti et al [38, 54, 55] recommend representing feather-CORT by unit of feather length (pg/mm), Lendvai et al [56] and Grunst et al [57] report that measurement error across samples is likely, and advise that feather-CORT be represented per unit of mass (pg/mg). In previous studies and across our samples (linear model, t = 30.704, p <0.0001), CORT standardized by unit of mass and length were highly correlated [57, 58]. We therefore chose to represent feather-CORT per unit mass (pg/mg).

For detailed methodology regarding assay validations, please refer to the supporting methodology (S2 Methods). Briefly, serially diluted, pooled feather extracts showed parallel displacement to CORT standard (r^2^ of curve constructed from pooled extract = 0.99) (S2 Fig). Recovery of exogenous CORT from spiked samples was 86.5% (± SE = 10.8%) (n = 5) and correlated with expected concentrations (r^2^ = 0.97) (S2 Fig). Intra- and inter-assay CV was 3.01% (± SE = 0.33), 11.7% (± SE = 2.89) respectively. Bortolotti et al (2009) [54] reported that feather segments with fault bars (areas of stunted or fragmented growth) exhibit higher deposition of CORT than adjacent segments of equal length with no fault bars. As a biologically relevant control, we therefore compared CORT deposition between feather sections with fault bars and adjacent sections without fault bars, as per Bortolotti et al (2009) [54]. Feather segments with fault bars contained significantly greater concentrations of deposited CORT by a one-tailed, paired Student’s T-Test (t = 1.826, df = 10, p = 0.049, n = 11) (S3 Fig).

### Statistical analysis

All analyses were conducted in the statistical program R, version 3.3.1 (R core, 2016). We first tested whether feather-CORT deposition differed between adults according to categorical group size, using a linear mixed effect model with Gaussian distribution in the package lme4 [59]. Here, categorical group size (atypically small, intermediately sized or atypically large) and sex were chosen as fixed effect predictor variables. Year sampled, territory and individual ID (to account for multiple samplings of the same individual) were initially chosen as random effects, with individual ID and territory nested within sample year. Sampling year and territory, however, explained little residual variance in our model (<0.001 and 0.066 respectively) and were therefore removed from analyses.

Feather-CORT (pg/mg) (the response variable) was log transformed to meet normality (Shapiro-Wilk test; p = 0.159, W = 0.966) and groups’ sizes were categorized according to deviation from the mean (*x* ± SD). Because optimal social group size can vary between sexes (ie. [11]), an interaction between sex and group size was initially included as a fixed effect predictor in our model, however this interaction was removed due to a non-significant effect (p = 0.531, F = 0.642) without altering significance of results. To test the robustness of our model to correctly reject a null hypothesis, we conducted a power analysis for n = 50 and r = 0.3 (moderate correlation) using the package ‘SIMR’ [60] (*β* = 0.78; lower CI = 0.64, upper CI = 0.88; AIC = 115.3). To assess overall effects, and not level-specific effects of each categorical predictor (sex and social group size) on log transformed feather-CORT, results of the linear mixed effect model were analyzed using the ‘anova’ function (R Base), followed by a between group comparison using a Tukey’s post-hoc test in the package lsmeans [61]. Alpha levels for analyses were set to 0.05.

## Results

### Feather corticosterone, sex and group size

**Fig 1:**
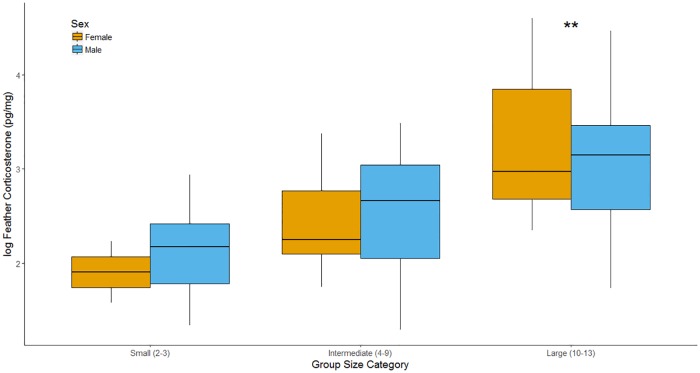
Log transformed feather corticosterone (pg/mg) of adult Smooth-billed Anis (*Crotophaga ani*) according to categorical social group size. Categorical group sizes determined by deviation from the mean (*x* (± SD). Yellow bars represent females and blue bars represent males. Median concentrations of corticosterone are shown with horizontal dark lines in boxes. Whiskers represent ± 1.5 times the interquartile range (distance from the fist to third quartile).

Our assay detected CORT in all feather segments (n = 50). Feather-CORT concentrations ranged from 3.64 to 99.67 pg/mg with a mean of 33.61 pg/mg (± SE = 7.89). Our model did not detect a significant effect of sex on log-transformed feather-CORT of adult Smooth-billed Anis (*x*_males_ = 17.73 pg/mg, ± SE = 2.65; *x*_females_ = 23.55 pg/mg, SE = 6.88) (Table 1) (S1 Fig). Categorical group size was a significant predictor of log feather-CORT (Table 1) and a Tukey’s post-hoc revealed significant differences between log feather-CORT from adults in intermediately sized and large groups, and between adults from small groups and large groups (Table 2), but not between adults from small and intermediately sized groups (Table 2) (Fig 1).

**Table 1:** Effect of categorical group size and sex on log transformed feather corticosterone (pg/mg) of adult Smooth-billed Anis (*Crotophaga ani*): Results of a linear mixed-effects model (n = 50).

Coefficient	Sum of Squares	df	F-value	p-value
Categorical Group Size	3.688	2	5.996	0.005*
Sex	2.00 x 10^-4^	1	6.00 x 10^-4^	0.981

asterisk (*) indicates significance at an alpha of 0.05.

**Table 2:** Post-hoc comparison of mean log-transformed feather corticosterone (pg/mg) according to categorical group sizes (n = 50).

Group Size Category Pair	Difference in Means	p-value
Small-Intermediate	-0.459	0.101
Small-Large	-1.088	0.002*
Intermediate-Large	-0.629	0.017*

Tukey’s method used for post-hoc analyses (n = 50). Asterisk (*) indicates significance at an alpha of 0.05.

## Discussion

Despite direct fitness benefits of group membership [36, 37], our analyses detect marked elevation of stress physiology in Smooth-billed Anis from atypically large social groups (Fig 1). Adults in atypically large groups showed significantly higher CORT deposited in rectrices than those in small and intermediately sized groups (Fig 1). Notably, we show no significant difference in feather-CORT between individuals from intermediate and atypically small groups, suggesting that the effects of social interaction on CORT mobilization are exaggerated in atypically large social groups, or buffered by benefits of group living in intermediately sized groups (ie reduced provisioning per capita, and shared predator vigilance) [36, 62, 63].

Elevated CORT deposition experienced by individuals in atypically large groups is most likely explained by reproductive competition. During breeding seasons, Smooth-billed Anis engage in intense at-the-nest competition for maternal representation in incubated clutches. Members of each sex engage in ovicide by egg tossing and burial, resulting in remarkable per capita egg losses that increase with social group size [35] (Quinn JS, personal observations). In both Smooth-billed Anis and closely related Groove-billed Anis (*Crotophaga sulcirostris*), eggs losses are generally compensated for by increased oviposition [35, 64]. Competitive interactions have long been known to elevate baseline glucocorticoids in socially living species [19, 28, 65–67] and competitive interactions behind egg burial and tossing are likely to follow suit. Indeed, Schmaltz et al [34] reported higher CORT deposited in yolk of Smooth-billed Ani eggs laid in multi-female groups when compared to single female pairs. Similarly, yolk CORT deposited in late laid eggs of multi-female groups (after ovicidal competition had taken place) was reportedly higher than that of early laid eggs [34], highlighting the influence of ovicide on stress physiology in female breeders. Aside from at-the-nest dynamics, enhanced competition for access to mates and resources in large social groups may also act to increase glucocorticoids in both sexes. Such patterns are common in social birds [33, 41, 68] and may outweigh benefits of social aggregation if chronic elevation becomes limiting to reproduction and/or survival [19, 28–31].

Interestingly, feather-CORT did not significantly differ between sexes (S4 Fig) and may suggest an equally shared costs of reproductive competition. In females, elevated deposition of feather-CORT in atypically large social groups likely reflects the physiological expenses compensatory oviposition. In previous studies, plasma CORT was dramatically elevated during egg-laying periods in female American Kestrels (*Falco sparverius*) [69] and in domestic laying hens (*Gallus gallus domesticus*) [70]. Similarly, nesting and egg-laying stages in female House Sparrows (*Passer domesticus*) coincided with elevated plasma CORT [71]. While all laying females are likely to experience elevations in baseline glucocortoids during breeding seasons, females in larger groups partake in compensatory egg production, therefore increasing annual oviposition and likely exacerbating physiological costs. Female Smooth-billed Anis from multi-female social groups have been shown to lay more eggs and deposit higher concentrations of CORT in yolk than those in single female groups [34, 35]—an action thought to occur by passive diffusion and therefore reflect circulating maternal CORT [72–74]. In males, heightened feather-CORT in atypically large social clusters is more likely explained by an exaggeration of intra-male competition for mating opportunities. Indeed, recent research [40] has suggested that sexual selection may be acting on male bill size—a trend supported by molecular analyses of extra-pair fertilizations [75]. Male-male competition has been reported to elevate plasma CORT in other socially monogamous bird species [76–79], which may be observable in patterns of feather-CORT deposition [38].

In all, we show that the use of feathers as a stable endocrine archive is sufficient to detect, and ask questions about CORT mobilization in response to social stressors. Interestingly, our results illustrate that feather-CORT deposition is highest in joint-nesting Smooth-billed Anis from atypically large social groups. We suspect that this elevation in baseline CORT is most likely due to reproductive competition, however, experimental brood reduction may aid in better elucidating causative relationships. Not surprisingly, atypically large groups are relatively infrequent in our field study (5 of 30 groups observed), but not rare. Given reduction of per capita reproductive success [35] and elevations of baseline CORT in large social groups, formation and maintenance of such may represent an evolutionarily unstable strategy if not offset by undescribed direct fitness benefits. We therefore recommend further investigation of the mechanisms driving group formation and maintenance in this species.

## Supporting information

S1 Fig Molt pattern of adult Smooth-billed Ani. (PDF)

S2 Fig Results of enzyme-linked immunosorbent assay validations. (PDF)

S3 Fig Corticosterone deposition in rectrix sections with and without fault bars. (PDF)

S4 Fig Corticosterone deposition in rectrices of male and female Smooth-billed Anis. (PDF)

S1 Methods Assessment of corticosterone and social group size with strict feather inclusion criteria. (PDF)

S2 Methods Enzyme-linked immunosorbent assay validations. (PDF)

S1 Table Effect of categorical group size and sex on adult feather corticosterone with strict feather selection criteria. (PDF)

S2 Table Post-hoc comparison of mean feather corticosterone between categorical group sizes with strict feather selection criteria. (PDF)
