# Supplementary material for: The effect of social group size on feather corticosterone in the co-operatively breeding Smooth-billed Ani (Crotophaga ani): An assay validation and analysis of extreme social living
Source: PLoS One. 2017 Mar 29;12(3):e0174650. doi: 10.1371/journal.pone.0174650 (PMC5371372; doi:10.1371/journal.pone.0174650)
Supplement: S1 Fig — (PDF) [file pone.0174650.s001.pdf]

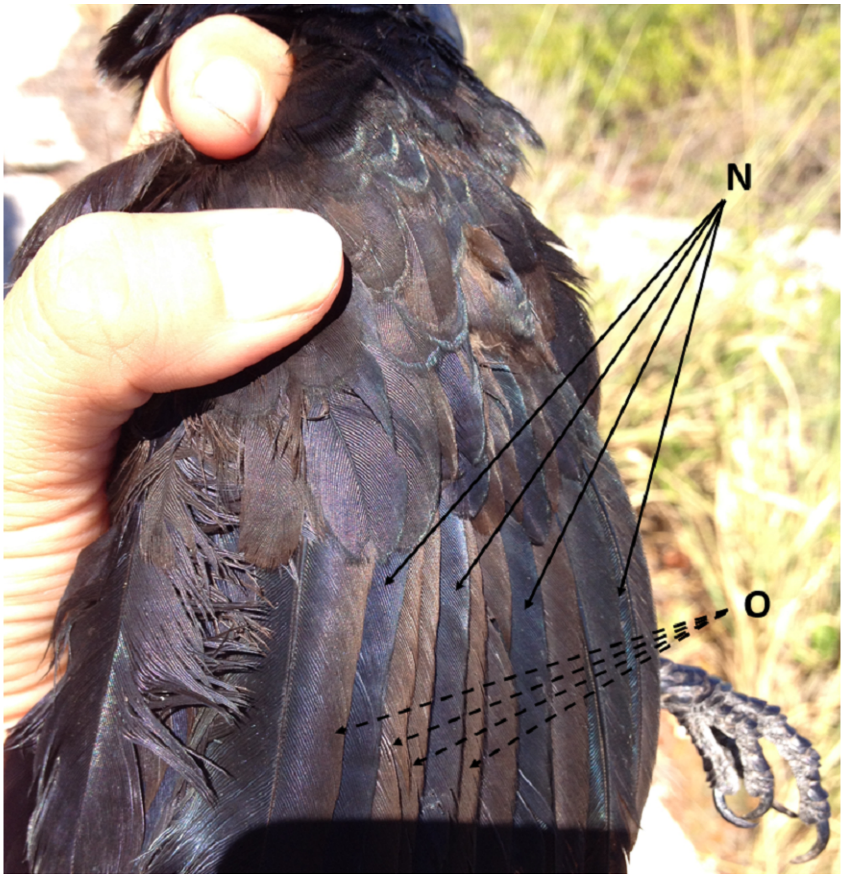

S1 Fig. Visible molt pattern of adult Smooth-billed Ani (*Crotophaga ani*) captured prior to the onset of breeding season. Primary and secondary flight feathers of a right wing shown from an adult captured in August, 2015. Newly grown feathers exhibit a marked iridescence (labeled 'N' and with solid arrows) and are distinct from feather grown in the previous season (labeled 'O' and with dashed arrows).
