# Supplementary material for: The effect of social group size on feather corticosterone in the co-operatively breeding Smooth-billed Ani (Crotophaga ani): An assay validation and analysis of extreme social living
Source: PLoS One. 2017 Mar 29;12(3):e0174650. doi: 10.1371/journal.pone.0174650 (PMC5371372; doi:10.1371/journal.pone.0174650)
Supplement: S2 Fig — (PDF) [file pone.0174650.s002.pdf]

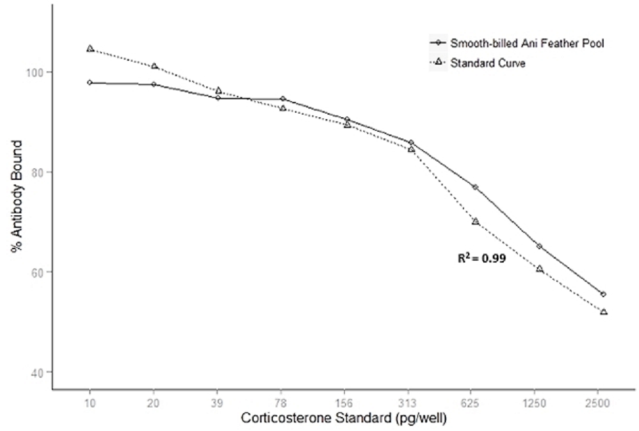

(a)

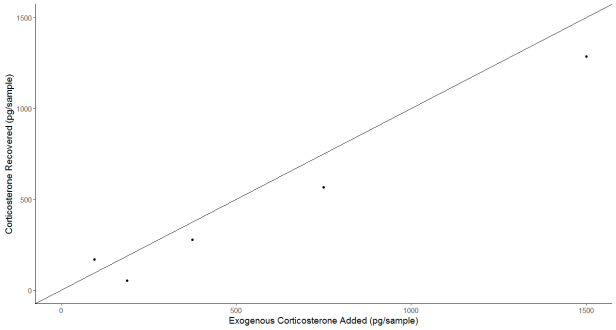

(b)

S2 Fig. Feather corticosterone assay validations.

(A) Parallel displacement of serially diluted, pooled Smooth-billed Ani feather extracts and corticosterone standard. Corticosterone standard dilutions are represented by open triangles and pooled feather extracts are represented by open circles. Trend lines are dashed and solid for corticosterone standard and pooled extracts respectively. (B) Expected recovery of exogenous corticosterone added to sample extracts drawn against observed corticosterone recovery (pg/sample).
