# Supplementary material for: The effect of social group size on feather corticosterone in the co-operatively breeding Smooth-billed Ani (Crotophaga ani): An assay validation and analysis of extreme social living
Source: PLoS One. 2017 Mar 29;12(3):e0174650. doi: 10.1371/journal.pone.0174650 (PMC5371372; doi:10.1371/journal.pone.0174650)
Supplement: S3 Fig — (PDF) [file pone.0174650.s003.pdf]

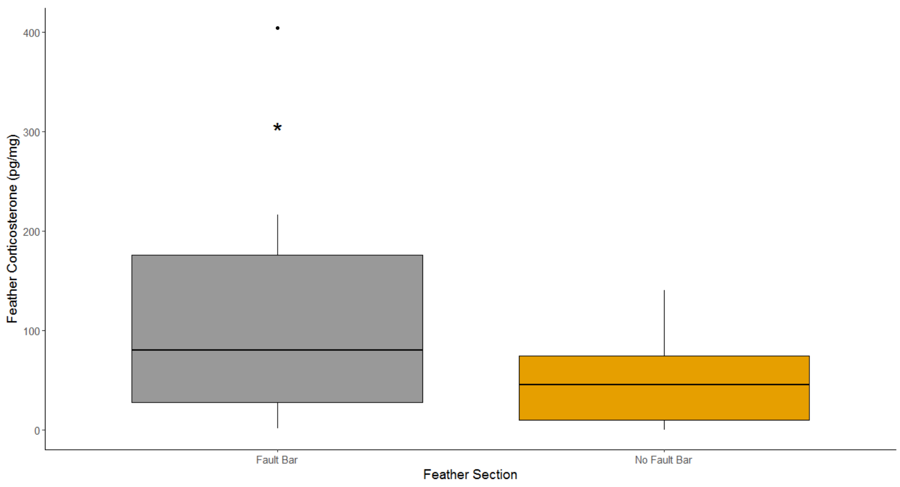

S3 Fig. Corticosterone deposition is in rectrix sections with and without the presence of fault bars.

Corticosterone (pg/mg) in tail feather segments (12 mm) containing fault bars, or no fault bars. Fault bar segments are represented in grey, and non-fault bar sections in yellow. Median concentrations of corticosterone are shown with horizontal dark lines in boxes. Whiskers represent ± 1.5 times the interquartile range (distance from the fist to third quartile).
