# Supplementary material for: The effect of social group size on feather corticosterone in the co-operatively breeding Smooth-billed Ani (Crotophaga ani): An assay validation and analysis of extreme social living
Source: PLoS One. 2017 Mar 29;12(3):e0174650. doi: 10.1371/journal.pone.0174650 (PMC5371372; doi:10.1371/journal.pone.0174650)
Supplement: S4 Fig — (PDF) [file pone.0174650.s004.pdf]

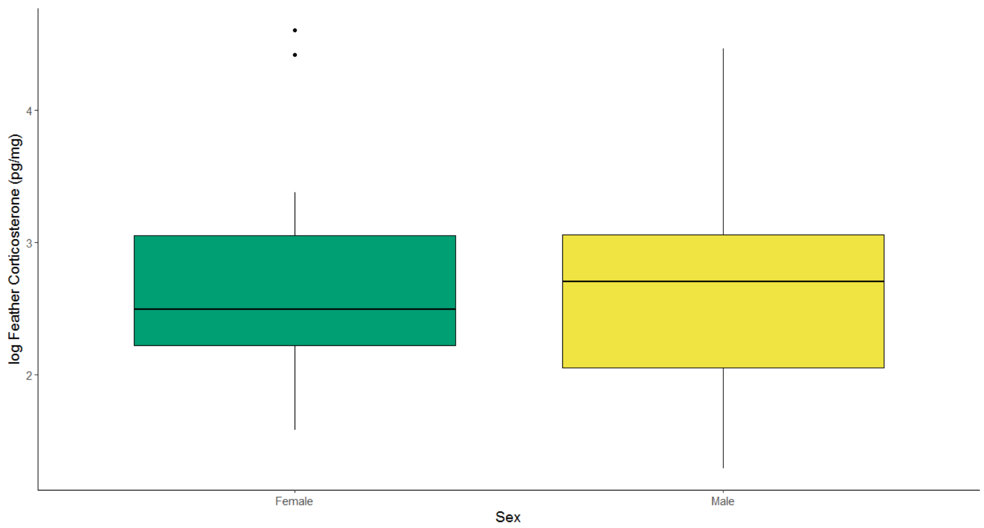

S4 Fig. Corticosterone (pg/mg) deposited in rectrices of female and male Smooth-billed Anis (*Crotophaga ani*). Females represented in green, and males in yellow. Median concentrations of corticosterone are shown with horizontal dark lines in boxes. Whiskers represent ± 1.5 times the interquartile range (distance from the fist to third quartile).
