# Supplementary material for: The effect of social group size on feather corticosterone in the co-operatively breeding Smooth-billed Ani (Crotophaga ani): An assay validation and analysis of extreme social living
Source: PLoS One. 2017 Mar 29;12(3):e0174650. doi: 10.1371/journal.pone.0174650 (PMC5371372; doi:10.1371/journal.pone.0174650)
Supplement: S1 Methods — (PDF) [file pone.0174650.s005.pdf]

S1 Methods

Assessment of Corticosterone and Social Group Size with Strict Feather Inclusion Criteria

We tested a correlation between social group size and deposition of CORT in rectrices of adult Smooth-billed Anis using a linear mixed-effects model in the R-package lme4 [59]. Here, categorical group size and sex were chosen as fixed effect predictors and sampling year, sampling territory and individual ID were included as random effects. Feather-CORT (pg/mg) was chosen as the Gaussian distributed response variable, but was first log-transformed to meet normality (Shapiro-Wilk test; $p = 0.159$, $W = 0.966$). Sampling year and territory explained little residual variance (<0.001 and 0.066 respectively), however, and were removed from the remainder of analyses.

To test the robustness of observed trends and ensure temporal localization within the breeding season, we removed feathers with mild fading ($n = 7$) and re-ran analyses as previously described. To assess an overall effect of a categorical predictor (social group size or sex) on feather-CORT deposition, we used the 'anova' function (R Base), then tested within categorical differences with a Tukey's post-hoc test [61]. Alpha levels for analyses were set to 0.05.
