# Supplementary material for: The effect of social group size on feather corticosterone in the co-operatively breeding Smooth-billed Ani (Crotophaga ani): An assay validation and analysis of extreme social living
Source: PLoS One. 2017 Mar 29;12(3):e0174650. doi: 10.1371/journal.pone.0174650 (PMC5371372; doi:10.1371/journal.pone.0174650)
Supplement: S2 Methods — (PDF) [file pone.0174650.s006.pdf]

S2 Methods

Assay Validations

Parallelism

We first tested parallel displacement of CORT-HRP by CORT standard and CORT extracted from feather samples. To do so, we created five pooled samples, each containing methanol-ether extracts from 10 randomly selected feather samples. Each pooled sample was concentrated 48-fold, then serially diluted twelve times in phosphate assay buffer. Serially diluted samples were then assessed in parallel with standard curves.

Assay Recovery

We assessed the accuracy of CORT detection in our assay by quantifying the percent recovery of exogenous CORT added to five randomly selected sample extracts. Each sample was concentrated 20-fold in assay buffer and spiked with a known concentration of CORT standard (1000, 500, 250, 125 and 62.5 pg). Samples were run according to assay protocols and percent recovery was assessed as prior.

Fault Bar Assessment

Punctuated stress in birds is capable of modulating feather growth and

can cause physical deformities in feather barbs and barbules known as fault bars [35]. Indeed, feather segments containing faults bars have been shown to contain heightened concentrations of deposited CORT [53]. We therefore tested the ability of our assay to detect expected differences in CORT deposition between adjacent feather segments (12 mm) with and without visible fault bars (within-feather) according to protocols described in Bortolotti et al [53].

Statistical Analyses

To first test whether pooled sample extracts mimicked displacement of CORT-HRP by CORT standard, we ran serial dilutions of CORT standard and sample extract side-by-side and plotted percent of antibody bound by concentration of CORT standard (S2 Fig A). A Pearson's r^2 was calculated for each curve. Next, we tested whether observed concentrations of CORT in spiked sample extracts were correlated with expected concentrations using a linear model (S2 Fig B). Finally, we tested whether feather sections containing fault bars had differing quantities of deposited CORT than adjacent sections without fault bars (S3 Fig). To do so, we compared sample means using a one-tailed, paired Student's T-Test.
