# Supplementary material for: The effect of social group size on feather corticosterone in the co-operatively breeding Smooth-billed Ani (Crotophaga ani): An assay validation and analysis of extreme social living
Source: PLoS One. 2017 Mar 29;12(3):e0174650. doi: 10.1371/journal.pone.0174650 (PMC5371372; doi:10.1371/journal.pone.0174650)
Supplement: S1 Table — (PDF) [file pone.0174650.s007.pdf]

S1 Table. Effect of categorical group size and sex on adult, log transformed feather corticosterone (pg/mg): results of a linear mixed-effects model with strict feather selection criteria (n = 43).

Coefficient	Sum of Squares	df	F-value	p-value
Categorical Group Size	3.845	2	5.589	0.008*
Sex	0.016	1	0.045	0.833

Individual ID alone included as a random effect in linear mixed-effects model. Feathers exhibiting mild fading removed from sample size. Asterisk (*) indicate statistical significance at alpha of 0.05.
