# Supplementary material for: The effect of social group size on feather corticosterone in the co-operatively breeding Smooth-billed Ani (Crotophaga ani): An assay validation and analysis of extreme social living
Source: PLoS One. 2017 Mar 29;12(3):e0174650. doi: 10.1371/journal.pone.0174650 (PMC5371372; doi:10.1371/journal.pone.0174650)
Supplement: S2 Table — (PDF) [file pone.0174650.s008.pdf]

S2 Table. Comparison of mean log-transformed feather corticosterone (pg/mg) between categorical group sizes: results of post-hoc comparison with strict feather selection criteria (n = 43).

Group Size Category Pair	Difference in Means	p-value
Small-Intermediate	-0.332	0.308
Small-Large	-1.082	0.007*
Intermediate-Large	-0.750	0.008*

Feathers exhibiting mild fading removed from sample size. Tukey's method used for post-hoc analyses. Asterix (*) indicates significance at an alpha of 0.05.
